# Intra Amniotic Administration of Raffinose and Stachyose Affects the Intestinal Brush Border Functionality and Alters Gut Microflora Populations

**DOI:** 10.3390/nu9030304

**Published:** 2017-03-19

**Authors:** Sarina Pacifici, Jaehong Song, Cathy Zhang, Qiaoye Wang, Raymond P. Glahn, Nikolai Kolba, Elad Tako

**Affiliations:** 1 Department of Animal Sciences, Cornell University, Ithaca, NY 14853, USA; sjp233@cornell.edu; 2 Department of Biological Sciences, Cornell University, Ithaca, NY 14853, USA; js2833@cornell.edu; 3 Division of Nutritional Sciences, Cornell University, Ithaca, NY 14853, USA; cz223@cornell.edu; 4 Department of Food Science and Technology, Cornell University, Ithaca, NY 14853, USA; yw696@cornell.edu; 5 USDA-ARS, Robert W. Holley Center for Agriculture and Health, Cornell University, Ithaca, NY 14853, USA; rpg3@cornell.edu (R.P.G.); nk598@cornell.edu (N.K.)

**Keywords:** raffinose, stachyose, brush border membrane, iron, prebiotics

## Abstract

This study investigates the effectiveness of two types of prebiotics—stachyose and raffinose—which are present in staple food crops that are widely consumed in regions where dietary Fe deficiency is a health concern. The hypothesis is that these prebiotics will improve Fe status, intestinal functionality, and increase health-promoting bacterial populations in vivo (*Gallus gallus*). By using the intra-amniotic administration procedure, prebiotic treatment solutions were injected in ovo (day 17 of embryonic incubation) with varying concentrations of a 1.0 mL pure raffinose or stachyose in 18 MΩ H_2_O. Four treatment groups (50, 100 mg·mL^−1^ raffinose or stachyose) and two controls (18 MΩ H_2_O and non-injected) were utilized. At hatch the cecum, small intestine, liver, and blood were collected for assessment of the relative abundance of the gut microflora, relative expression of Fe-related genes and brush border membrane functional genes, hepatic ferritin levels, and hemoglobin levels, respectively. The prebiotic treatments increased the relative expression of brush border membrane functionality proteins (*p* < 0.05), decreased the relative expression of Fe-related proteins (*p* < 0.05), and increased villus surface area. Raffinose and stachyose increased the relative abundance of probiotics (*p* < 0.05)*,* and decreased that of pathogenic bacteria. Raffinose and stachyose beneficially affected the gut microflora, Fe bioavailability, and brush border membrane functionality. Our investigations have led to a greater understanding of these prebiotics’ effects on intestinal health and mineral metabolism.

## 1. Introduction

Iron (Fe) deficiency is the most common nutrient deficiency worldwide, affecting between 30% and 40% of the world’s population [1,2]. Those who suffer from this condition can experience fatigue, cognitive impairment, and death [3]. The prevalence of Fe deficiency in these geographical regions can be attributed to the populations’ consumption of low-diversity plant-based diets including cereals and legumes, which contain low amounts of bioavailable Fe as well as compounds such as polyphenols that further inhibit Fe absorption [4,5]. Dietary Fe deficiency and other dietary mineral deficiencies stem from a lack of essential nutrients in staple food crops, and thus health sectors have turned to various agricultural interventions as potential solutions. A form of intervention that shows great promise is biofortification. Biofortification refers to the use of traditional breeding practices to bring about significant increases in bioavailable micronutrients in the edible portions of food crops [6,7]. Once biofortified, the seedlings of staple foods crops can be distributed to farmers who are already experienced in growing these particular crops. In fact, biofortified crops are found to have a multitude of agronomic benefits for farmers due to their high micronutrient stores, including disease resistance, improved seed viability, greater seedling vigor, lower seeding rate requirements, and faster root establishment—all of which lead to increased productivity relative to the original seedlings [7]. Another sustainable aspect of this solution is that once biofortified varieties are grown, they will not continue to require government attention or funding [7].

However, a major challenge associated with biofortification of staple food crops—namely cereal grains and legume seeds—in developing regions is that they contain factors such as polyphenols and phytic acid that inhibit the absorption of Fe [8]. When these crops are biofortified via conventional breeding, there is the potential for these inhibitory factors to increase along with Fe [9,10,11]. Despite containing inhibitory factors, cereal grains and legumes also carry other substances, referred to as promoters, which have the potential to counteract the effects of these inhibitory factors. Thus, one prospective solution to the aforementioned dilemma is to increase the content of these promoter substances to counteract the negative effects of the inhibitory factors such as polyphenols [9]. One of the most notable of these promoter substances is the prebiotic [9,12].

Prebiotics are polysaccharides that have been shown to enhance the growth and activities of probiotics, or beneficial gut microflora. These compounds are capable of surviving acidic and enzymatic digestion in the small intestine, and thus can be fermented by probiotics that reside in the colon/cecum [6]. The fermentation of prebiotics by probiotics leads to the production of short-chain fatty acids, which lower intestinal pH, inhibiting the growth of potentially pathogenic bacterial populations and improving the absorption of minerals such as Fe [7]. Raffinose and stachyose were chosen as the prebiotics for investigation in this study, since they are found in high concentrations in lentils and chickpeas [8], which are staple crops consumed by populations in which Fe deficiency is a health concern [5]. Previously, we demonstrated the effects of the wheat prebiotics arabinoxylans and fructans on intestinal probiotics [12] as a potential approach to improving Fe bioavailability in staple food crops and gut health.

In the current study, raffinose and stachyose effects were studied in vivo by utilizing the poultry model (*Gallus gallus*). The broiler chicken is a fast-growing animal with sensitivities to dietary deficiencies of trace minerals such as Fe [13], and is very receptive to dietary manipulations [9,12,13,14]. Additionally, there is >85% homology between gene sequences of human and chicken intestinal genes such as DMT1, DcytB, ZnT1, and Ferroportin [15]. Thus, one objective of this study is to assess the effects of intra-amniotic raffinose and stachyose administration on Fe status in vivo (*Gallus gallus*), an animal model that has been used to investigate the physiological effects of various nutritional solutions [16,17]. Specifically, the expression of Fe metabolism-related genes (DMT1, the major Fe intestinal transporter; DcytB, Fe reductase; and Ferroportin, the major intestinal enterocyte Fe exporter), in the duodenum (the major Fe absorption site). The second objective in using this model is to assess the effects of raffinose and stachyose on brush border membrane (BBM) development and functionality using biomarkers for BBM absorptive ability such as the relative expressions of aminopeptidase (AP), sucrase isomaltase (SI), and sodium glucose cotransporter-1 (SGLT1), as well as the surface areas of the intestinal villi. The third objective is to evaluate the effects of the intra-amniotic administration of these prebiotics on intestinal bacterial populations by measuring the relative abundances of probiotic health-promoting populations bacteria such as *Bifidobacterium* and *Lactobacillus* versus those of potentially pathogenic bacteria such as *E. coli* and *Clostridium*.

The goal in investigating these effects is to determine whether raffinose and stachyose may be candidates for biofortification in staple food crops. If they demonstrate the ability to improve Fe status, BBM functionality, and intestinal bacterial populations, breeding lentils and chickpeas for increased stachyose and raffinose content may potentially eliminate the need for exogenous Fe supplementation by increasing the bioavailability of Fe in these crops. The distribution of biofortified crop seedlings would serve as a sustainable means of combating malnutrition in developing regions where dietary Fe deficiency is common.

## 2. Materials and Methods

### 2.1. Animals and Design

Cornish cross-fertile broiler chicken eggs (*n* = 120) were obtained from a commercial hatchery (Moyer’s chicks, Quakertown, PA, USA). The eggs were incubated under optimal conditions at the Cornell University Animal Science poultry farm incubator.

### 2.2. Intra-Amniotic Administration

All animal protocols were approved by Cornell University Institutional Animal Care and Use committee (ethic approval code: 2007-0129). Pure stachyose and raffinose in powder form were separately diluted in 18 MΩ H_2_O to determine the concentrations necessary to maintain an osmolarity value of less than 320 Osm to ensure that the chicken embryos would not be dehydrated upon injection of the solution. This intra-amniotic administration procedure followed that of Tako et al. [12]. On day 17 of embryonic incubation, eggs containing viable embryos were weighed and divided into 6 groups (*n* = 12) with an approximately equal weight distribution. The intra-amniotic treatment solution (1 mL per egg) was injected with a 21-gauge needle into the amniotic fluid, which was identified by candling. After injection, the injection sites were sealed with cellophane tape. The six groups were assigned as follows: 1. 5% stachyose (in 18 MΩ H_2_O); 2. 10% stachyose (in 18 MΩ H_2_O); 3. 5% raffinose (in 18 MΩ H_2_O); 4. 10% raffinose (in 18 MΩ H_2_O); 5. 18 MΩ H_2_O; 6. non-injected. Eggs were placed in hatching baskets such that each treatment was equally represented at each incubator location.

### 2.3. Tissue Collection

On the day of hatch (day 21), birds were euthanized by CO_2_ exposure. The small intestines, ceca, blood, and livers were quickly removed from the carcasses and placed in separate tubes for storage. The samples were immediately frozen in liquid nitrogen and then stored in a −80 °C freezer until analysis.

### 2.4. Isolation of Total RNA

Total RNA was extracted from 30 mg of small intestine (duodenal) tissue using Qiagen RNeasy Mini Kit. RNA was quantified by absorbance at 260–280 nm. Integrity of the 28S and 18S rRNA was verified by 1.5% agarose gel electrophoresis followed by ethidium bromide staining.

### 2.5. Gene Expression Analysis

As was previously described [9,12,13], RT-PCR was carried out with primers chosen from the fragments of chicken duodenal tissues. After the completion of PCR, the results were run under gel electrophoresis on 2% agarose gel stained with ethidium bromide for separation of the target genes (DMT1, Ferroportin, DcytB, AP, SI, SGLT1). Quantity One 1D analysis software (Bio-Rad, Hercules, CA, USA) was utilized to quantify the resulting bands. Highly conserved tissue-specific 18S rRNA was used as internal standard to normalize the results.

### 2.6. Bacterial Analysis

As was previously described [18,19,20], the contents of the ceca were placed into a sterile 50 mL tube containing 9 mL of sterile phosphate-buffered saline (PBS) and homogenized by vortexing with glass beads. Debris was removed by centrifugation. For DNA purification, the pellet was treated with lysozyme. The bacterial genomic DNA was isolated using a Wizard Genomic DNA purification kit. Primers for *Lactobacillus*, *Bifidobacterium*, *Clostridium*, and *E. coli* were designed according to previously published data by Zhu et al. in 2002 [19]. The universal primers—which identify all known strains of bacteria in the intestine—were prepared with the invariant region in the 16S rRNA of bacteria, and were used as internal standard to normalize the results. The DNA samples underwent PCR, and the amplified results were loaded on 2% agarose gel stained with ethidium bromide and underwent electrophoresis for separation. Then, the bands were quantified using Quantity One 1-D analysis software (Bio-Rad, Hercules, CA, USA). Abundance of individual bacterial gene expression was measured relative to the universal primer product, where the total bacteria equaled 100%.

### 2.7. Assessment of Liver Ferritin

As was previously described [9,14], the collected liver samples were treated similarly to the procedures described in a previous study by Passaniti et al. in 1989 [21]. Approximately 0.25 g of liver sample was diluted into 0.5 mL of 50 mM Hepes buffer (pH 7.4) and homogenized on ice using an UltraTurrax homogenizer at maximum speed (5000× *g*) for 2 min. Each homogenate was subjected to heat treatment for 10 min at 75 °C to aid isolation of ferritin. The samples were immediately cooled on ice for 30 min after heat treatment, centrifuged at 13,000× *g* for 30 min until a clear supernatant was obtained, and the pellet containing insoluble denatured proteins was discarded. Native polyacrylamide gel electrophoresis was utilized for separation technique. Six percent separating gel and 5% stacking gel were prepared for the procedure. A constant 100 V voltage was administered throughout the process. The resulting gels were then treated with two specific stains: potassium ferricyanide (K_3_Fe(CN)_6_)—a stain specific for Fe—and Coomassie blue G-250 stain, specific for protein in general. The Fe-stained bands represented ferritin levels, and were compared to the corresponding bands in the Coomasie-stained gel to calculate relative abundance of ferritin (ferritin-to-total-protein ratio). Gels were scanned by using the Bio-Rad densitometer and measured using the Quantity-One 1-D analysis program (Bio-Rad, Hercules, CA, USA). Horse spleen ferritin was used as a standard to calibrate ferritin/Fe concentrations. Ferritin saturation levels were measured by calculating relative percentage of Fe present in the protein to the maximum number of Fe atoms that can be present per molecule of ferritin (approximately 4500 Fe atoms) [22].

### 2.8. Blood Analysis and Hb Measurements

Blood was collected using micro-hematocrit heparinized capillary tubes (Fisher Scientific, Waltham, MA, USA). Blood Hb concentrations were determined spectrophotometrically using the cyanmethemoglobin method (H7506-STD, Pointe Scientific Inc., Canton, MI, USA) following the kit manufacturer's instructions.

### 2.9. Morphological Examination of the Intestinal Villi

As was previously described [17], intestinal samples (duodenal region as the main intestinal Fe absorption site) at day of hatch from each treatment were fixed in fresh 4% (*vol*/*vol*) buffered formaldehyde, dehydrated, cleared, and embedded in paraffin. Serial sections were cut at 5 µm and placed on glass slides. Sections were deparaffinized in xylene, rehydrated in a graded alcohol series, stained with hematoxylin and eosin, and examined by light microscopy. Morphometric measurements of villus height and width were performed with an Olympus light microscope using EPIX XCAP software. Villus surface area was calculated from villus height and width at half height.

### 2.10. Goblet Cell Diameter

Morphometric measurements of goblet cell diameter were performed with an Olympus light microscope using EPIX XCAP software.

### 2.11. Statistical Analysis

Results were analyzed by one-way multiple analysis of variance (MANOVA) using the JMP software (SAS Institute Inc., Cary, NC, USA). Differences between treatments were compared by Tukey’s test, and values were statistically different at *p* < 0.05 (values in the text are means ± SEM).

## 3. Results

### 3.1. Intestinal Content Bacterial Genera- and Species-Level Analysis

**Figure 1:**
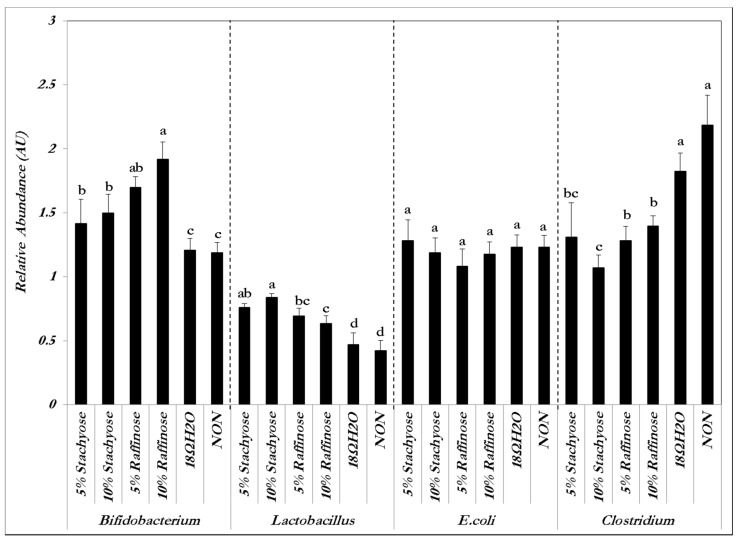
Genera and species-level bacterial populations (AU) from cecal contents measured on the day of hatch. Values are means ± SEM, *n* = 8. Per bacterial category, treatment groups not indicated by the same letter are significantly different (*p* < 0.05).

The relative abundance of both *Bifidobacterium* and *Lactobacillus*—which are known to be probiotics—significantly (*p* < 0.05) increased in the presence of both concentrations of stachyose and raffinose. The relative abundance of *E. coli* did not significantly (*p* > 0.05) increase or decrease in the presence of the prebiotic treatment solutions compared to the controls. *Clostridium*’s relative abundance significantly (*p* < 0.05) decreased in the presence of both concentrations of stachyose and raffinose compared to the controls (Figure 1). These results indicate that stachyose and raffinose improved gut health by promoting the survival of probiotics and limiting the existence of potentially pathogenic bacterial populations. The presence of these probiotics was expected to give rise to an increase in short-chain fatty acid production and an increase in Fe solubility, and in turn, Fe bioavailability.

### 3.2. BBM Functional Genes

**Figure 2:**
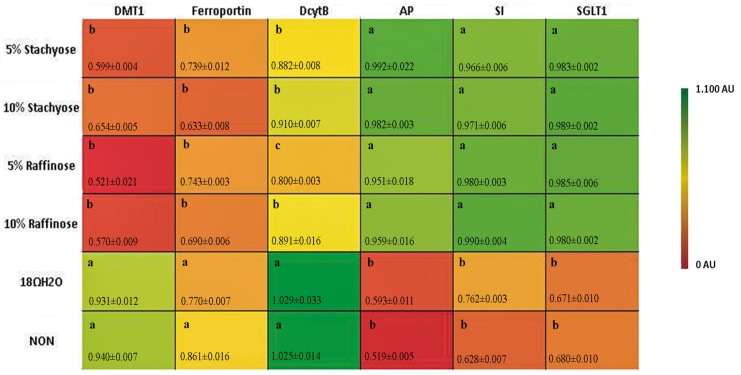
Duodenal mRNA expression (in AU) of measured brush border membrane (BBM) functional and Fe metabolism genes on the day of hatch. Values are means ± SEM, *n* = 8. Standard errors are represented by vertical bars. Per gene, treatments groups not indicated by the same letter are significantly different (*p* < 0.05). AP: aminopeptidase; SI: sucrase isomaltase; SGLT1: sodium glucose cotransporter-1.

The relative expressions of AP, SI, and SGLT1 were all significantly (*p* < 0.05) up-regulated in the presence of both concentrations of stachyose and raffinose (Figure 2). The up-regulation of the expression of BBM functional genes signifies increased absorptive ability of the BBM, which indicates improved functionality and gut health [23].

### 3.3. Fe Metabolism Genes

The relative expressions of DcytB, DMT1, and ferroportin were all significantly (*p* < 0.05) down-regulated in the presence of both concentrations of stachyose and raffinose (Figure 2). The down-regulation of these genes is in turn suggested to be an indicator of Fe-replete conditions. This is a potential biomarker for improved Fe status.

### 3.4. Cecum-to-Body-Weight Ratio

The cecum-to-bodyweight ratios were significantly (*p* < 0.05) higher in the prebiotic treatment groups compared to the controls. The ceca of subjects that received stachyose and raffinose increased, indicating an increase in their content of bacterial populations (Table 1).

### 3.5. Morphometric Data for Villi

The villus surface areas significantly (*p* < 0.05) increased in the presence of both concentrations of stachyose and raffinose (Table 2). This serves as a mechanical measurement of BBM absorptive ability and improvement in BBM functionality and gut health by indicating that the introduction of stachyose and raffinose enhanced proliferation of enterocytes.

### 3.6. Goblet Cell Diameters

The goblet cell diameters significantly (*p* < 0.05) increased in the presence of both concentrations of stachyose and raffinose (Table 2).

### 3.7. Liver Ferritin and Hb

There were no significant (*p* > 0.05) differences in ferritin or Hb values between treatment groups. The lack of a significant difference in these physiological measurements of Fe status between groups is posited to be because there was not enough Fe in the environment to create a significant change (Table 3).

## 4. Discussion

This study utilized the *Gallus gallus* model, as it is a fast growing animal with relatively high mineral requirements, and hence can develop deficiency considerably quickly [13]. Previous studies have shown that intra-amniotic administration is useful for investigating the effects of specific nutrients at particular stages of intestinal development [16,17,24]. According to Ludwiczek et al., intracellular Fe concentrations play a role in regulating Fe absorption into the enterocytes and are beneficial to bacteria within the cecum [25].

There was an increased abundance of both *Lactobacillus* (*p* < 0.05) and *Bifidobacterium* (*p* < 0.05), and a decrease in *Clostridium* in the cecal contents of the birds that received the prebiotic treatments. However, there were no significant differences for *E. coli* between the groups (*p* > 0.05), and likewise a lack of significant differences within and between the raffinose and stachyose groups (*p* > 0.05) of same bacterial species (Figure 1). As previously mentioned, *Lactobacillus* and *Bifidobacterium* are known probiotics, whereas *Clostridium* is a potentially pathogenic genus and *E. coli* can be either pathogenic or beneficial, depending on the strain [20,26,27]. *Lactobacillus* and *Bifidobacterium* both produce short chain fatty acids (SCFA), potentially increasing Fe bioavailability and reducing the abundance of pathogenic bacteria that utilize dietary Fe in the colon [28,29]. Other prebiotic compounds such as inulin have been shown to enhance the proliferation of selected beneficial colonic microflora [26,30]. *Bifidobacterium* and *Lactobacillus* have an advantage over other intestinal microorganisms due to their β-1,2-glycosidase activity that allows them to break down prebiotics, resulting in their proliferation and possibly leading to greater SCFA production [18,19,20]. Therefore, it is reasonable to suspect that the intra-amniotic administration of stachyose and raffinose may improve Fe status by inducing a more efficient Fe uptake and intestinal transferring. In addition to the more efficient Fe uptake, the cecum-to-bodyweight ratios were higher in the prebiotic treatment groups versus the control groups (*p* < 0.05), indicating that the cecal content of chickens that received intra-amniotic prebiotic solutions was greater than those that did not. This observation is used as an indicator for a potential increase in cecal bacterial populations (Table 1) [12].

In addition, the expressions of duodenal (the major Fe absorption site) Fe metabolism-related proteins (DMT1, the major Fe intestinal transporter; DcytB, Fe reductase; and Ferroportin, the major intestinal enterocyte Fe exporter), was decreased in treatment groups receiving the raffinose and stachyose solutions versus controls (*p* < 0.05) (Figure 2). These results are comparable to those of a previous study conducted on late-term broiler embryos and hatchlings, in which treatment groups with improved Fe status expressed significantly less Fe-related proteins [31]. The results suggest that the increased Fe bioavailability led to Fe-sufficient conditions, meaning an increase in Fe metabolism-related transporters and enzymes were not required (as a compensatory mechanism). Furthermore, there was no significant differences (*p* > 0.05) between the two raffinose administered groups in DMT1 and ferroportin; whereas DcytB actually showed a significant decrease (*p* < 0.05) in the 10% versus 5% raffinose (Figure 2). One reasonable explanation is that SCFA produced from bacteria increases Fe^3+^ solubility, resulting in more bioavailable Fe that led to the decreased DcytB expression [16].

The significant increase in the expression of the BBM functionality genes (AP, SI, SGLT1, *p* < 0.05) is in agreement with the morphometric measurements, indicating that intra-amniotic administration of raffinose and stachyose improved BBM functionality and potentially enhanced the absorptive and digestive capacity of the villi (Table 2). As is evident in the current study, it was previously suggested that dietary prebiotics increase probiotics’ butyrate production, which may lead to cellular (enterocyte) proliferation. It was also demonstrated that birds that received intra-amniotic administration of carbohydrates developed villi with greater surface areas compared to the untreated birds [32]. Scholz-Ahrens et al. (2007) and Preidis et al. (2012) support these results by concluding that some of their respective prebiotics increase cellular proliferation, which causes the increase in villi surface area [33,34]. Additionally, we also measured an increase in goblet cell diameter, which suggests an increased production of mucus that coats the intestinal lumen and effects bacterial composition and function [35,36].

Overall, the up-regulation of BBM functional proteins, down-regulation of Fe metabolism proteins, in addition to the increase in the relative abundance of beneficial probiotics, intestinal villi surface area, and goblet cell diameters, suggest that stachyose and raffinose are promising in their potential for improving Fe status and BBM functionality.

## 5. Conclusions

The current research validates the need for future studies that could incorporate these plant origin prebiotics in long-term feeding trials. The potential breeding of staple food crops such as lentils and chickpeas for increased stachyose and raffinose contents may serve as a sustainable means of combating malnutrition in developing regions, which is a strategy that has been proven effective in wheat [12]. This study served as a preliminary step to provide a greater understanding of the way that various prebiotics can alleviate dietary deficiencies.

## Figures and Tables

**Table 1:** Cecum-to-body weight ratio (%). Values are means ± SEM, *n* = 12. ^a,b^ Treatment groups not indicated by the same letter are significantly different (*p* < 0.05).

Treatment Group	Cecum/Body Weight Ratio (%)
5% Stachyose	1.67 ± 0.21 ^a^
10% Stachyose	1.63 ± 0.18 ^a^
5% Raffinose	1.83 ± 0.20 ^a^
10% Raffinose	1.55 ± 0.13 ^a^
18ΩH_2_O	1.35 ± 0.08 ^b^
Non-injected	1.22 ± 0.07 ^b^

**Table 2:** Effect of intra-amniotic administration of experimental solutions on the duodenal small intestinal villus surface area (mm^2^) and goblet cells diameter (µm). Values are means ± SEM, *n* = 6. ^a–e^ Treatment groups not indicated by the same letter are significantly different (*p* < 0.05).

Treatment Group	Villus Surface Area (mm^2^)	Goblet Cell Diameter (µm)
5% Stachyose	459.2 ± 32.09 ^a,b^	18.2 ± 0.143 ^c^
10% Stachyose	493.8 ± 10.31 ^a^	19.1 ± 0.152 ^b^
5% Raffinose	467.5 ± 35.55 ^a,b^	22.5 ± 0.180 ^a^
10% Raffinose	425.2 ± 24.04 ^b,c^	19.5 ± 0.156 ^b^
18ΩH_2_O	384.4 ± 14.16 ^c^	15.4 ± 0.123 ^d^
Non-injected	353.1 ± 13.24 ^c^	13.2 ± 0.105 ^e^

**Table 3:** Liver Ferritin protein amounts (AU) and blood hemoglobin (Hb) concentrations (g/dL). Values are means ± SEM, *n* = 10. ^a^ Treatment groups not indicated by the same letter are significantly different (*p* < 0.05).

Treatment Group	Ferritin (AU)	Hb (g/dL)
5% Stachyose	0.48 ± 0.09 ^a^	10.7 ± 0.54 ^a^
10% Stachyose	0.48 ± 0.08 ^a^	11.1 ± 0.55 ^a^
5% Raffinose	0.47 ± 0.10 ^a^	10.5 ± 0.68 ^a^
10% Raffinose	0.48 ± 0.09 ^a^	11.0 ± 0.56 ^a^
18ΩH_2_O	0.47 ± 0.09 ^a^	10.4 ± 0.42 ^a^
Non-injected	0.47 ± 0.07 ^a^	10.3 ± 0.65 ^a^

## Abbreviations

Fe	iron
Hb	hemoglobin
DMT1	divalent metal transporter 1
DcytB	duodenal cytochrome B
AP	amino peptidase
SI	sucrose isomaltase
SGLT-1	sodium glucose transporter 1
BBM	brush border membrane
